# Antimicrobial Resistance in Veterinary Medicine and Public Health

**DOI:** 10.3390/ani12233253

**Published:** 2022-11-23

**Authors:** Paola Roncada, Bruno Tilocca

**Affiliations:** Department of Health Sciences, University Magna Græcia of Catanzaro, 88100 Catanzaro, Italy

## Editorial

Animal productions, and populations, have been rapidly expanding over the last decades, forcing the ever-closer coexistence of human beings and domestic animals on our yet “narrow” planet. In addition to the damage to crops, natural resources, and loss of animal biodiversity, the increased contact occurring between humans and animals greatly supports the transmission of zoonotic diseases. This, in turn, enables the onset and the rapid diffusion of antimicrobial resistance (AMR) traits across the three sectors of life (human, animal, and the environment). In this view, veterinary medicine and the surrounding environment play an important part in the public health threat, in line with the One-Health concept [1].

Major clinically relevant bacteria for virulence and antibiotic resistance traits are the so-called ESKAPE pathogens, an acronym referring to *Enterococcus faecium*, *Staphylococcus aureus*, *Klebsiella pneumoniae*, *Acinetobacter baumanii*, *Pseudomonas aeruginosa*, and *Enterobacter* spp. Antibiotics misuse (e.g., following improper prescription, overuse in the agricultural field and usage as growth promoting agents) results in a selective pressure stimulating the bacterial adaptation mechanisms which, unavoidably, include the increasing trend to chromosomal mutations and/or a higher tendency to acquire exogenous nucleic acids (i.e., free DNA, plasmids, etc.) seeking for more favourable (adapted) phenotypes. Antibiotic-resistant bacteria are subsequently selected, and their clonal expansion amplifies the AMR traits and their diffusion, both vertically to daughter cells and horizontally to further recipient cells [1,2].

The co-occurrence of mixed microbial communities in the same ecological niches favours the massive diffusion of the genetically determined resistance traits at both intra- and inter-specific levels. Evidence of interkingdom transmission of the antimicrobial traits has also been documented, underlining the impact of this phenomenon across boundaries and its potential invalidating effect on human and veterinary antibiotic-based therapies. In this light, further efforts are desirable in understanding the diverse facets of antimicrobial resistance, ranging from assessing the geographic diffusion of diverse AMR traits to how these dynamic fluxes evolve in time and space to reach the molecular mechanisms (novel and past) employed for the inactivation of antibiotic therapies [2].

This Special Issue harbours 11 published research articles summarizing studies on diverse aspects of the antimicrobial resistance phenomenon, providing significant levels of innovation and knowledge suggestive of further research routes. All the contributions of this Special Issue emphasize the need for the cautious assessment of the circulation of AMR traits in the clinical and veterinary fields, including the assessment of the contributions by microorganisms of food and environmental origin in the dissemination of antimicrobial resistance traits across the three sectors of life. Kwon et al. investigated extended-spectrum cephalosporin (ESC)-resistant *Salmonella* spp. of chicken origin to assess the transferability to humans of beta-lactamase gene-harbouring plasmid in vitro and in vivo. Warningly the study provides evidence on the ease of dissemination of the AMR traits between bacteria (*Salmonella–Escherichia*) in the case of coexistence, as it is common in microbial community settings, even in the absence of antibiotic-mediated selective pressure, providing a clear glimpse on the role of the food chain in the dissemination of the AMR from a One-Health perspective [3]. Another study by Kwon and colleagues [4] assessed the prevalence of *Campylobacter* spp. over the whole chicken production process. The study highlights the hot spot of AMR dissemination in the chicken meat production process, underlining that resistance to fluoroquinolones was the most frequently observed form of resistance in their sample.

Mtemisika and colleagues [5] surveyed the presence of resistant *Escherichia coli* in pigs and poultry populations reared in Mwanza, Tanzania, according to standard operating procedures and international guidelines. This study highlights that although different *E. coli* phenotypes are harboured in the animal’s intestine, AMR traits have been recorded in both host types.

Piras et al. [6] investigated the persistence of *S. aureus* strains as means of biofilm production, a very well-known burden in food, farm and clinical contexts. The study detected mechanisms related to the control of catabolites, the production of proteins with moonlighting activities and the detoxification of compounds with antimicrobial activities, suggesting potential biomarkers/metabolic routes to be targeted to prevent and/or mitigate this phenomenon. Another study by Piras and co-workers [7] focused on the effective resistance traits of the milk-associated microbial community, focusing on the microbial protein repertoire. Proteins are indeed effective actors in the resistance mechanisms, and their study provides a detailed glimpse into the microbial dynamics and the metabolic influence that milk microbiota members exert on each other.

Overton et al. [8] employed a metabolomic approach to investigate the effect of multiple antibiotic molecules on *Salmonella typhimurium* cultured from various hosts. Interestingly, the authors highlight a significant modulation in the metabolic profiles, suggesting metabolomics as an innovative approach for the quick evaluation of resistance traits against multiple antibiotics. 

The study of Abd El-Aziz et al. [9] provides the first survey into the virulence traits, antimicrobial and biocide resistance and epidemiological typing of *Streptococcus uberis* isolated from bovine clinical mastitis in dairy farms of diverse hygienic interventions in Egypt. The survey describes the prevalence of *S. uberis* infections and the genes involved in its virulence and antimicrobial resistance in relation to the hygienic conditions of the dairy farm, underlining the importance of a combined intervention while facing the dissemination of pathogens and their resistance traits.

A further study by Abd El-Aziz and colleagues [10] investigated, for the first time, class 1 integrons and associated gene cassettes among pan-drug-resistant (PDR), extensively drug-resistant (XDR) and multidrug-resistant (MDR) *Campylobacter spp*. Although no pan-susceptible isolates were found, the study describes the detection of multiple resistance traits, in addition to providing the very first identification of a putative phage tail tape measure protein which is indicative of a possible *Campylobacter*–bacteriophage interaction, resulting in the consequent spread of the resistance trait via horizontal gene transfer.

Kang and colleagues [11] focused on environmental mastitis-causing enterococci as an emerging cause of nosocomial infections of relevance due to their antimicrobial resistance traits. The survey underlined a dramatically high proportion of the bulk tank milk-derived enterococci as the vector of resistance traits against single- and multi-antimicrobial drugs.

Zou and collaborators [12] described the prevalence and molecular features of extraintestinal pathogenic *Escherichia coli* (ExPEC). In addition, the study observes the presence of the plasmid-borne colistin resistance gene along with an additional carbapenemase gene in some isolates. Altogether, their findings suggest that healthy chickens can serve as a potential reservoir for multidrug-resistant ExPEC isolates.

Interestingly, this Special Issue also includes a study by Nazish et al. [13] on antinematode resistance of *Haemonchus contortus*. It suggests the use of *Comamonas* spp. and *Pseudomonas weihenstephanesis* as biological control agents to be employed as alternatives to synthetic anthelmintic compounds, showing a significant mortality rate against the parasite with little-to-no selective pressure triggering the onset and diffusion of the antimicrobial resistance traits. 

Altogether, the above research articles deal with several aspects of antimicrobial resistance, providing an enlightening view and suggestive approaches for future research lines aimed at tackling antimicrobial resistance in the diverse aspects of the life sciences. Both expert scientists and readers approaching this fascinating field can benefit from the cutting-edge analysis of an impressive range of data from diverse sample types, and we hope you enjoy it.

## References

[1] Murugaiyan J., Kumar P.A., Rao G.S., Iskandar K., Hawser S., Hays J.P., Mohsen Y., Adukkadukkam S., Awuah W.A., Jose R.A.M. (2022). Progress in Alternative Strategies to Combat Antimicrobial Resistance: Focus on Antibiotics. Antibiotics.

[2] Palma E., Tilocca B., Roncada P. (2020). Antimicrobial Resistance in Veterinary Medicine: An Overview. Int. J. Mol. Sci..

[3] Kwon B.-R., Wei B., Cha S.-Y., Shang K., Zhang J.-F., Jang H.-K., Kang M. (2021). Characterization of Extended-Spectrum Cephalosporin (ESC) Resistance in *Salmonella* Isolated from Chicken and Identification of High Frequency Transfer of *bla*_CMY-2_ Gene Harboring Plasmid In Vitro and In Vivo. Animals.

[4] Kwon B.-R., Wei B., Cha S.-Y., Shang K., Zhang J.-F., Kang M., Jang H.-K. (2021). Longitudinal Study of the Distribution of Antimicrobial-Resistant *Campylobacter* Isolates from an Integrated Broiler Chicken Operation. Animals.

[5] Mtemisika C.I., Nyawale H., Benju R.J., Genchwere J.M., Silago V., Mushi M.F., Mwanga J., Konje E., Mirambo M.M., Mshana S.E. (2022). Epidemiological Cut-Off Values and Multidrug Resistance of *Escherichia coli* Isolated from Domesticated Poultry and Pigs Reared in Mwanza, Tanzania: A Cross-Section Study. Animals.

[6] Piras C., Di Ciccio P., Soggiu A., Greco V., Tilocca B., Costanzo N., Ceniti C., Urbani A., Bonizzi L., Ianieri A. (2021). *S. aureus* Biofilm Protein Expression Linked to Antimicrobial Resistance: A Proteomic Study. Animals.

[7] Piras C., Greco V., Gugliandolo E., Soggiu A., Tilocca B., Bonizzi L., Zecconi A., Cramer R., Britti D., Urbani A. (2020). Raw Cow Milk Bacterial Consortium as Bioindicator of Circulating Anti-Microbial Resistance (AMR). Animals.

[8] Overton J.M., Linke L., Magnuson R., Broeckling C.D., Rao S. (2022). Metabolomic Profiles of Multidrug-Resistant *Salmonella* Typhimurium from Humans, Bovine, and Porcine Hosts. Animals.

[9] El-Aziz N.A., Ammar A., El Damaty H., Elkader R.A., Saad H., El-Kazzaz W., Khalifa E. (2021). Environmental *Streptococcus uberis* Associated with Clinical Mastitis in Dairy Cows: Virulence Traits, Antimicrobial and Biocide Resistance, and Epidemiological Typing. Animals.

[10] El-Aziz N.K.A., Ammar A.M., Hamdy M.M., Gobouri A.A., Azab E., Sewid A.H. (2020). First Report of *aacC5-aadA7Δ4* Gene Cassette Array and Phage Tail Tape Measure Protein on Class 1 Integrons of *Campylobacter* Species Isolated from Animal and Human Sources in Egypt. Animals.

[11] Kang H., Yoon S., Kim K., Lee Y. (2021). Characteristics of High-Level Aminoglycoside-Resistant *Enterococcus faecalis* Isolated from Bulk Tank Milk in Korea. Animals.

[12] Zou M., Ma P.-P., Liu W.-S., Liang X., Li X.-Y., Li Y.-Z., Liu B.-T. (2021). Prevalence and Antibiotic Resistance Characteristics of Extraintestinal Pathogenic *Escherichia coli* among Healthy Chickens from Farms and Live Poultry Markets in China. Animals.

[13] Nazish A., Fozia, Khattak B., Khan T.A., Ahmad I., Ullah R., Bari A., Asmari M., Mahmood H., Sohaib M. (2021). Antinematode Activity of Abomasum Bacterial Culture Filtrates against *Haemonchus contortus* in Small Ruminants. Animals.

